# Effectiveness of securing central venous catheters with topical tissue adhesive in patients undergoing cardiac surgery: a randomized controlled pilot study

**DOI:** 10.1186/s12871-021-01282-0

**Published:** 2021-03-08

**Authors:** Naruemol Prachanpanich, Sunthiti Morakul, Napanont Kiatmongkolkul

**Affiliations:** Department of Anesthesiology, Faculty of Medicine, Ramathibodi Hospital, Mahidol University, Bangkok, 10400 Thailand

**Keywords:** Topical tissue adhesive, Central venous catheter, Cardiac surgery, Intensive care unit

## Abstract

**Background:**

Central venous catheters (CVCs) play an important role during cardiac surgery. Topical tissue adhesives form a thin film of coating that becomes bound to keratin in the epidermis. The advantage of this “super glue” lies in its antimicrobial activity. This study aimed to evaluate fixation of CVCs with topical tissue adhesive in patients (prone to bleed) undergoing cardiac surgery regarding its ability to reduce the incidence of pericatheter leakage.

**Methods:**

This randomized controlled trial included 150 patients > 15 years of age who were (1) scheduled to undergo elective cardiac surgery, (2) required CVC insertion at the internal jugular vein, and (3) scheduled for transfer postoperatively to the cardiac intensive care unit. We randomly assigned patients to a topical tissue adhesive group (TA) or a standard control group (SC). The primary outcome was a change in dressing immediately postoperatively due to pericatheter blood oozing. Secondary outcomes were the number of dressings, total dressings per catheter day, and composite outcome of catheter failure within 3 days. Both intention-to-treat and per-protocol analyses were performed. Seven patients violated the protocol (three TA patients and four SC patients).

**Results:**

Regarding the primary outcome, the SC group exhibited a significantly increased incidence of dressing change immediately postoperatively due to pericatheter leakage compared with the TA group in both the intention-to-treat analysis (5.33% vs 18.67%, RR 0.25 [95% CI 0.08 to 0.79], *P* = 0.012) and the per-protocol analysis (5.56% vs 16.90%, RR 0.289 [95% CI 0.09 to 0.95], *P* = 0.031). No significant differences were noted in the number of dressings, total dressings per catheter day, or composite outcome of catheter failure within 3 days between the two groups. Multiple logistic regression analysis was performed to adjust baseline characteristics that were different in the per-protocol analysis. The results showed that the risk ratio of immediate postoperative dressing change in TA patients was 0.25 compared to the SC group ([95% CI 0.07 to 0.87], *P* = 0.029) in the per-protocol analysis.

**Conclusion:**

The use of a topical tissue adhesive can reduce the incidence of immediate postoperative pericatheter blood oozing.

**Trial registration:**

TCTR20180608004, retrospectively registered on June 06, 2018.

## Background

Central venous catheters (CVCs) play a role in critically ill patients who often require central venous pressure monitoring, pulmonary artery catheterization, transvenous cardiac pacing, and drug administration, among other techniques. However, CVC insertion is an invasive procedure that may lead to any one of several complications, such as pneumothorax, hematoma, pericatheter leakage, localized infection, and catheter-related bloodstream infection (CRBSI). The goal of CVC utilization is to facilitate medical treatment without complications.

From an anesthetic aspect, CVC is generally selected for patients at high operative risk, such as those scheduled to undergo cardiac surgery. These patients tend to have pericatheter leakage because most of them are at risk of bleeding from antiplatelet/anticoagulant medication exposure or due to cardiopulmonary bypass, and even massive blood loss is possible. Once pericatheter leakage occurs, it increases the risk of infection—both localized and CRBSI—due to oozing blood acting as a medium that allows bacterial overgrowth [1].

Currently, topical tissue adhesives, or “super glues” [e.g., octyl cyanoacrylate (Dermabond®) and n-butyl cyanoacrylate (Histoacryl®)], are widely used in the surgical field [2, 3]. A long-chain cyanoacrylic glue is available that can form a permanent thin film coating that binds to keratin in the epidermis, remaining intact for 5–10 days, after which it naturally sloughs off with the epithelia [4]. The advantages of these glues are not only that they can tightly close a surgical wound (as an alternative to sutures or staples) but also that they prevent leakage and control bleeding.

Clinical studies have demonstrated that Dermabond maintained a catheter-to-nerve apposition in a patient under continuous interscalene nerve block [5] and decreased pericatheter local anesthetic leakage in a patient undergoing continuous perineural infusion [6]. Dermabond is also an effective barrier to microbial penetration by Gram-positive and Gram-negative motile and nonmotile species [7]. In contrast, although Dermabond provides antimicrobial activity against Gram-positive bacteria, it is ineffective against fungi and Gram-negative bacteria [8–10]. Another report described the use of histoacryl to secure an epidural catheter and CVC [11, 12]. Moreover, Dermabond significantly increased the pull-out force in an experimental study, which may be an effective securement technique for intravascular catheters [9].

The Dermabond dressing was firmly adherent but easily peeled off when the catheter needed to be removed. There have been some reports of abrasion, especially when the catheter is removed soon after administration [4, 9]. There have also been infrequent reports that topical tissue adhesives were associated with allergic contact dermatitis [13–15]. With these positive and negative reports in mind, we decided to explore whether fixation of a CVC with Dermabond might decrease the rate of pericatheter leakage.

## Methods

This randomized controlled clinical trial adheres to CONSORT guidelines and was performed on 150 patients in Ramathibodi Hospital from May 2018 to March 2019. The study was approved by the Ramathibodi Ethics Committee (ID 03–61-10, Date of approval: April 25, 2018). Patients who had been selected were informed by the researchers about the research methods. After being informed, patients gave their written consent. Informed consent was obtained from all participants in the trial. The trial was registered at thaiclinicaltrials.org (TCTR20180608004, Date of registration: June 06, 2018). The first case enrolled in the study was on May 15, 2018. A late registration did not experience any changes in the study protocol.

### Participants

Eligible patients included those > 15 years of age who were scheduled to undergo elective cardiac surgery, required CVC insertion at the internal jugular vein in the operating room and were scheduled for transfer postoperatively to the cardiac intensive care unit (ICU). Exclusion criteria included refusal to participate, the presence of signs of infection or sepsis, and/or a history of atopic disease.

### Randomization/intervention/blinding

Demographic characteristics, including the risk of bleeding tendency, were collected at enrollment. The bleeding tendency is defined in Table 1.

**Table 1:** Causes of bleeding tendency in patients in this study

Cause	Definition
**Antiplatelet**	
Acetylsalicylic acid (ASA)	Within 7 days before operation
Clopidogrel	Within 5 days before operation
**Anticoagulant**	
Warfarin	Within 5 days before operation
Enoxaparin	Within 12 h before operation
Rivaroxaban, Apixaban, Dabigatran	Within 3 days before operation
**Thrombocytopenia**	Platelet count < 140,000/mL
**Coagulopathy**	
Prolonged thrombin time (TT)	> 14 s
Prolonged partial thromboplastin time (PTT)	> 35 s
Prolonged prothrombin time (PT)	> 13.5 s
Prolonged PT-international normalized ratio	> 1.2

We randomly assigned patients into two groups via block-of-four randomization sequences using a computer-generated randomization website (http://www.sealedenvelope.com). Allocation concealment was performed using sealed envelopes. Blinding was not possible in this study design. The standard control (SC) group comprised patients who underwent routine CVC insertion by an anesthesiologist or anesthesia department resident under supervision. The wound was covered with 1 × 1-cm gauze and a transparent dressing with Tegaderm (not Dermabond). In the topical tissue adhesive (TA) group, the patients underwent routine CVC insertion (same as that for the SC group), but Dermabond was applied to the catheter at its insertion site before applying 1 × 1-cm gauze and Tegaderm (as in the SC group). The CVC was fixed by suturing both groups with 3–0 nylon or silk sutures.

All patients underwent general anesthesia and standard monitoring, including electrocardiography, pulse oximetry, noninvasive blood pressure measurements, end-tidal carbon dioxide monitoring, and invasive monitoring, such as arterial blood pressure measurements.

The technique, time period of CVC insertion(s) (before vs. after anesthesia induction), number of CVCs per location, or medications for inducing and maintaining the patient under general anesthesia depended on the decisions of the clinician who assessed the patient preoperatively and attended him or her during the maintenance phase of the operation. The CVC insertion technique included an ultrasonography-guided or landmark technique. The technique of insertion was decided by an anesthesiologist in charge who did not take participate in patient allocation. After the operation was completed, the clinician who was accountable for the patient to the end of the operation evaluated the CVC insertion site to determine whether there was any pericatheter bleeding before transferring the patient to the cardiac ICU. If there was some pericatheter bleeding, which defined here as the total wetness of the 1 × 1-cm gauze, whether to change the dressing was decided by the clinician who had no role in the study design or data analysis. Data were recorded during the operative period and continued thereafter, and the postoperative bleeding risk was monitored. After admission to the cardiac ICU, the patient’s CVC was assessed and attended to by the ward nurse and clinicians to evaluate any pericatheter bleeding and, if so, how much. Patients were also monitored for any signs of infection.

**Fig. 1:**
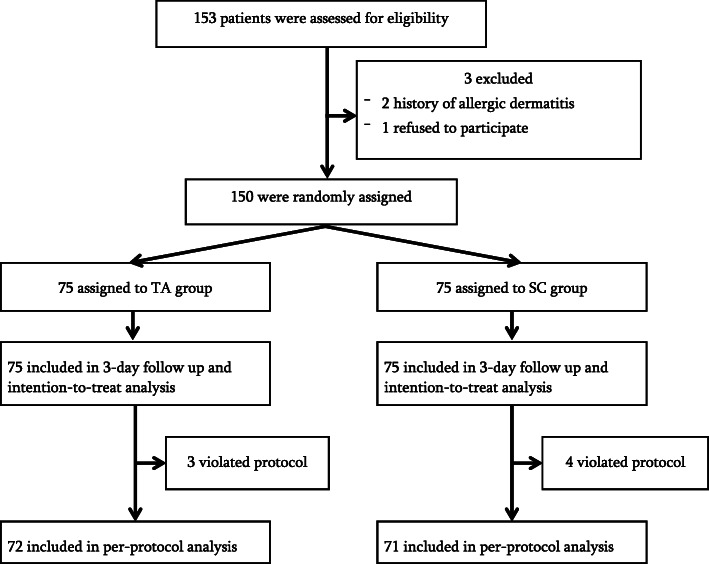
Flow chart of the study’s progression

The primary outcome of the study was having to change the dressing immediately postoperatively due to pericatheter leakage. The secondary outcomes were the number of dressings used, total dressings per catheter day, and a composite outcome of catheter failure within 3 days, which included failed fluid infusion via the catheter, hematoma development, and/or infection. A flowchart of the study’s progression is shown in Fig. 1.

### Statistical analysis

The sample size was calculated based on a recommendation for a pilot randomized trial [16]. The sample size of 150 for standardizing the extra small effect sizes (≤0.1) is recommended for a trial designed with 90% power and two-sided 5% significance.

Intention-to-treat and per-protocol analyses were performed. The intention-to-treat analysis included all patients who fulfilled the randomization process. For the per-protocol analysis, we excluded patients who switched treatment during the trial before achieving the primary outcome (*n* = 7). There were no missing data.

Descriptive analysis was performed using means, medians, standard deviations, and interquartile ranges for continuous variables and number counts with percentages for categorical variables. Categorical variables were compared using the *x*^2^ test. The normal distribution of each data set was confirmed using the Kolmogorov–Smirnov test. The t test was used to compare data with a parametric distribution. If the data were not normally distributed, the Mann–Whitney U test was employed. Statistical analysis was performed using SPSS 20.0 software (IBM SPSS Statistics for Windows, Version 20.0; (IBM Corp., Armonk, NY, USA). A significance level of *P* < 0.05 was used for all statistical analyses. If the results or baseline characteristics exhibited a significant difference, a multiple logistic regression was conducted to determine whether it was an independent predictor associated with immediate postoperative dressing change.

## Results

Altogether, 150 patients, including 80 (53%) males and 70 (46%) females, were randomized into two equal groups. As depicted in Table 2, most of the baseline variables and demographic characteristics exhibited no difference between the two groups except the number of catheters and LPRC transfusions in the per-protocol analysis. The patients in the TA group had many more double catheters than those in the SC group (61.11% vs 42.25%, *P* = 0.024). In contrast, the leukocyte-poor red blood cell (LPRC) transfusion rate was significantly increased in the SC group compared with the TA group by per-protocol analysis (55.56% vs 71.83%, *P* = 0.043).

**Table 2:** Baseline characteristics

Characteristic	Intention-to-treat analysis			Per-protocol analysis		
	TA (***n*** = 75)	SC (***n*** = 75)	***P***	TA (***n*** = 72)	SC (***n*** = 71)	***P***
Sex, male, n (%)	42 (56.00)	38 (50.67)	0.513	40 (55.56)	34 (47.89)	0.359
Age (yrs), mean ± SD	63.75 ± 13.75	63.35 ± 14.89	0.860	63.99 ± 13.75	63.54 ± 15.20	0.876
BMI (kg/m^2^), mean ± SD	24.69 ± 4.63	23.68 ± 4.43	0.173	24.70 ± 4.68	23.67 ± 4.53	0.181
ASA classification, n (%)			0.479			0.676
III	21 (28.00)	25 (33.33)		21 (29.17)	23 (32.39)	
IV	54 (72.00)	50 (66.67)		51 (70.83)	48 (67.61)	
Preoperative bleeding risk, n (%)	32 (42.67)	40 (53.33)	0.191	31 (43.06)	38 (53.52)	0.210
Anesthesia time (min), mean ± SD	356.27 ± 92.01	383.4 ± 117.22	0.135	355.90 ± 93.70	385.35 ± 119.65	0.110
Operations, n (%)			0.484			0.345
Valve surgery	33 (44.00)	39 (52.00)		31 (43.06)	38 (53.52)	
CABG	35 (46.67)	26 (34.67)		34 (47.22)	23 (32.39)	
CABG + Valve surgery	4 (5.33)	6 (8.00)		4 (5.56)	6 (8.45)	
Other	3 (4.00)	4 (5.33)		3 (4.17)	4 (5.63)	
On CPB, n (%)	58 (77.33)	58 (77.33)	> 0.999	55 (76.39)	56 (78.87)	0.722
Heparin dose (mg), mean ± SD	219.47 ± 94.13	220.61 ± 106.27	0.660	218.33 ± 95.42	221.56 ± 106.56	0.570
Protamine dose (mg), mean ± SD	287.93 ± 132.98	293.53 ± 151.72	0.613	286.25 ± 135.17	294.72 ± 153.47	0.530
No. of catheters, n (%)			0.050			0.024*
Single catheter	30 (40.00)	42 (56.00)		28 (38.89)	41 (57.75)	
Double catheters	45 (60.00)	33 (44.00)		44 (61.11)	30 (42.25)	
Insertion attempts, n (%)			0.827			0.373
> 2 Attempts	13 (17.33)	12 (16.00)		13 (18.06)	9 (12.68)	
1–2 Attempts	62 (82.67)	63 (84.00)		59 (81.94)	62 (87.32)	
Insertion technique, n (%)			0.618			0.453
Landmark technique	29 (38.67)	32 (42.67)		27 (37.50)	31 (43.66)	
Ultrasonography-guided technique	46 (61.33)	43 (57.33)		45 (62.50)	40 (56.34)	
Intraoperative LPRC transfusion, n (%)	42 (56.00)	53 (70.67)	0.062	40 (55.56)	51 (71.83)	0.043*
Intraoperative FFP transfusion, n (%)	51 (68.00)	57 (76.00)	0.275	48 (66.67)	56 (78.87)	0.101
Intraoperative LPPC transfusion, n (%)	52 (69.33)	58 (77.33)	0.268	49 (68.06)	56 (78.87)	0.143
Intraoperative cryoprecipitate transfusion, n (%)	9 (12.00)	15 (20.00)	0.181	8 (11.11)	15 (21.13)	0.103
Intraoperative autologous blood transfusion, n (%)	55 (73.33)	56 (74.67)	0.852	54 (75.00)	54 (76.06)	0.883
Postoperative bleeding risk, n (%)	72 (96)	74 (98.67)	0.620	69 (95.83)	70 (98.59)	0.620

*ASA* American Society of Anesthesiologists, *BMI* body mass index, *CPB* cardiopulmonary bypass, *FFP* fresh-frozen plasma, *LPPC* leukocyte-poor platelet concentrates, *LPRC* leukocyte-poor red blood cells, *SC* standard control, *SD* standard deviation, *TA* topical tissue adhesive

*P < 0.05

Regarding the primary outcome of the study, the SC group experienced a significantly higher incidence of immediate postoperative dressing changes due to pericatheter leakage than the TA group in both the intention-to-treat analysis (5.33% vs 18.67%, RR 0.25, [95% CI 0.08 to 0.79], *P* = 0.012) and the per-protocol analysis (5.56% vs 16.90%, RR 0.29, [95% CI 0.09 to 0.95], *P* = 0.031).

The differences in the number of dressings, the total dressings per catheter day, and the composite outcome of catheter failure within 3 days were not statistically significant as shown in Table 3. Two patients in the TA group developed a complication during the first postoperative day. One experienced fluid leakage within the soft tissues around the catheter, and the other had a problem with fluid administration via the catheter.

**Table 3:** Results

Parameter	Intention-to-treat analysis				Per-protocol analysis			
	TA (75)	SC (75)	***Relative risk (95%CI)***	***P***	TA (***n*** = 72)	SC (***n*** = 71)	***Relative risk (95%CI)***	***P***
Dressing changes immediately postoperatively, n (%)	4 (5.33)	14 (18.67)	0.25 (0.08–0.79)	0.012*	4 (5.56)	12 (16.90)	0.29 (0.09–0.95)	0.031*
Total dressings (number), median (IQR)	2 (1–2)	2 (1–2)		0.361	2 (1–2)	2 (1–2)		0.403
Total dressing/catheter-day, median (IQR)	0.5 (0.3–0.6)	0.3 (0.3–0.6)		0.661	0.5 (0.3–0.6)	0.3 (0.3–0.6)		0.892
Composite outcome of catheter failure, n (%)	2 (2.67)	–		0.497	2 (2.78)	–		0.497

*CI* confidence interval, *IQR* interquartile range, *SC* standard control, *TA* topical tissue adhesive

*P < 0.05

Multiple logistic regression analysis (Table 4) was performed to adjust baseline characteristics and demonstrated that the patients in the TA group who had topical tissue adhesive applied to the CVC were at significantly lower risk of immediate postoperative dressing change due to pericatheter leakage compared with the SC group (RR 0.25, [95% CI 0.07 to 0.87], *P* = 0.029) in the per-protocol analysis.

**Table 4:** Multiple logistic regression model for predicting immediately postoperative dressing change (per-protocol analysis)

Independent variables	Relative risk (95%CI)	***P***
Topical adhesive applied ^a^	0.25 (0.07–0.87)	0.029^*^
Double catheters ^b^	1.12 (0.38–3.30)	0.843
No Intraoperative LPRC transfusion	1.80 (0.60–5.40)	0.295

*CI* confidence interval, *LPRC* leukocyte-poor red blood cells

^a^ Referenced to standard control group (SC)

^b^ Referenced to single catheter

^*^P < 0.05

The number needed to treat (NNT) was calculated. The results were eight patients in the intention-to-treat analysis and nine in the per-protocol analysis.

## Discussion

This study showed that the use of a topical tissue adhesive can reduce the incidence of immediate postoperative pericatheter blood oozing even though TA group patients had many more catheters than SC group patients. This might be described by the property of producing a thin film coating at the insertion site [4]. Consistent with this finding, Gurnaney et al. [6] found that Dermabond may be useful for preventing pericatheter local anesthetic leakage after continuous perineural infusions, which could reduce exposure of the catheter to the environment.

As previously mentioned, a lower incidence of pericatheter leakage should decrease the incidence of both localized and systemic infections. However, our study did not reveal a difference in the infection rate, which might be explained by the time course of this complication. Specifically, it takes a longer time to develop an infection than within the 3 postoperative days that we chose for this study. However, this trial was designed as a pilot study, so the question of whether the number of infections is reduced when using this technique should be further investigated in a future study.

Other limitations of this study were that our protocol did not control the CVC insertion technique or that of the suturing method to secure the CVC. We also did not assess the primary outcome using an objective measurement, but this is less problematic because the study was designed to be a pragmatic study. We could not assign the same evaluator to assess every operation due to the limitations of health care personnel and high-risk patients. Hence, the evaluator who had no benefit from the study might have been the same clinician who inserted the CVC and applied a sterile dressing. As mentioned, there was no possibility of effective blinding. The third limitation was the frequency of dressing changes that were interrupted by the hospital protocol, which is to care for CVCs with the aim of preventing CRBSI. However, this protocol applied to both study groups; thus, the factor might have minimal influence.

Finally, our study did not evaluate patient satisfaction or the cost-effectiveness of applying a topical tissue adhesive to secure the CVC. Furthermore, our study results demonstrate benefit, especially in patients undergoing cardiac surgery who are prone to bleeding. However, the results of our study show beneficial outcomes only in immediate pericatheter leakage. For these reasons, our study results might not be applicable generally, and further study is needed.

## Conclusions

We recommend that a topical tissue adhesive be applied to the CVC in patients who are at risk of perioperative bleeding, such as those undergoing cardiac surgery or those who use an anticoagulant or antiplatelet medication. The aim is to reduce the incidence of pericatheter leakage immediately postoperatively, which would lead to changing a sterile dressing without an increased incidence of complications.

## Data Availability

The datasets used and/or analysed during the current study are available from the corresponding author on reasonable request.

## Abbreviations

CRBSI: Catheter-related bloodstream infection
CVC: Central venous catheter
ICU: Intensive care unit
LPRC: Leukocyte-poor red blood cells
SC: Standard control (group)
TA: Topical tissue adhesive (group)

## References

[1] O'Grady NP, Alexander M, Burns LA (2011). Guidelines for the prevention of intravascular catheter-related infections. Clin Infect Dis.

[2] Rickard CM, Marsh N, Webster J (2015). Securing all intravenous devices effectively in hospitalised patients—the SAVE trial: study protocol for a multicentre randomised controlled trial. BMJ Open.

[3] Ayyıldız SN, Ayyıldız A (2017). Cyanoacrylic tissue glues: biochemical properties and their usage in urology. Turk J Urol.

[4] Klein SM, Nielsen KC, Buckenmaier CC, Kamal AS, Rubin Y, Steele SM (2003). 2-Octyl cyanoacrylate glue for the fixation of continuous peripheral nerve catheters. Anesthesiology..

[5] Auyong DB, Cantor DA, Green C, Hanson NA (2017). The effect of fixation technique on continuous interscalene nerve block catheter success: a randomized, double-blind trial. Anesth Analg.

[6] Gurnaney H, Kraemer FW, Ganesh A (2011). Dermabond decreases pericatheter local anesthetic leakage after continuous perineural infusions. Anesth Analg.

[7] Bhende S, Rothenburger S, Spangler DJ, Dito M (2002). In vitro assessment of microbial barrier properties of Dermabond topical skin adhesive. Surg Infect.

[8] Wilkinson JN, Chikhani M, Mortimer K, Gill SJ (2008). The antimicrobial effect of Histoacryl skin adhesive. Anaesthesia..

[9] Simonova G, Rickard CM, Dunster KR, Smyth DJ, McMillan D, Fraser JF (2012). Cyanoacrylate tissue adhesives: effective securement technique for intravascular catheters: in vitro testing of safety and feasibility. Anaesth Intensive Care.

[10] Rushbrook JL, White G, Kidger L, Marsh P, Taggart TF (2014). The antibacterial effect of 2-octyl cyanoacrylate (Dermabond®) skin adhesive. J Infect Prev.

[11] Wilkinson JN, Sheikh N, Jayamaha J (2007). Tissue adhesive as an alternative to sutures for securing central venous catheters. Anaesthesia..

[12] Wilkinson J, Fitz-Henry J (2008). Securing epidural catheters with Histoacryl glue. Anaesthesia..

[13] Jagannathan N, Hallman M (2010). Complications associated with 2-octyl cyanoacrylate (Dermabond): considerations for the anesthesiologist. J Clin Anesth.

[14] Nakagawa S, Uda H, Sarukawa S (2018). Contact dermatitis caused by Dermabond advanced use. Plast Reconstr Surg Glob Open.

[15] Liu T, Wan J, McKenna RA, Jackson OA, Treat JR (2019). Allergic contact dermatitis caused by Dermabond in a paediatric patient undergoing skin surgery. Contact Dermatitis.

[16] Whitehead AL, Julious SA, Cooper CL, Campbell MJ (2016). Estimating the sample size for a pilot randomised trial to minimise the overall trial sample size for the external pilot and main trial for a continuous outcome variable. Stat Methods Med Res.

